# Variation in the quantity of venom produced by snakes at the national center for production and strategic public health goods and the need to update antivenom estimations

**DOI:** 10.17843/rpmesp.2025.424.15158

**Published:** 2025-12-12

**Authors:** Gualberto Marcas, Gilmer Solis-Sanchez, Flor Fuentes, Leavit López

**Affiliations:** 1 Centro Nacional de Producción y Bienes Estratégicos de Salud Pública, Instituto Nacional de Salud, Lima, Peru. Centro Nacional de Producción y Bienes Estratégicos de Salud Pública Instituto Nacional de Salud Lima Peru; 2 Centro de Evaluación de Tecnologías en Salud, Instituto Nacional de Salud, Lima, Peru. Centro de Evaluación de Tecnologías en Salud Instituto Nacional de Salud Lima Peru

Mr. Editor. Snakebite poisoning is a public health problem driven by various factors such as deforestation in the jungle, the intrusion of mining into previously virgin areas, and seasonal crops in tropical ecosystems, among others. The Peruvian herpetofauna is particularly diverse, and most snakebite poisonings are caused by *Bothrops atrox* (80%), with poisonings by bites from *Bothrops pictus*, *Bothrops barnetti*, *Lachesis muta*, and *Crotalus durissus*, among others, being reported less frequently ^1^.

In Peru, the management of poisonings has been based for decades on antivenoms produced by the National Center for Production and Strategic Public Health Goods (CNPB) of the National Institute of Health, using serum from horses injected with venom from snakes from the Oswaldo Meneses serpentarium. This center houses viperids of the three aforementioned genera, whose venom is extracted through a standardized process ^2^.

As it is a neglected disease, studies on this subject are scarce. Currently, the Technical Standard for the Prevention and Treatment of Accidents by Poisonous Animals from 2005 ^3^ establishes recommendations for the antivenom doses to be used in patient management. It states that specific treatment with antivenom serum in Peru is carried out with antibothropic, anticrotalic, and antilachetic serum with neutralization doses of 25 to 40 mg of venom per vial ^4^. The potency of the antivenom is verified through neutralization assays (ED₅₀) in mice and lethal dose 50 (LD₅₀) assays. Only after confirming that the antivenom neutralizes 2.5 mg of venom per mL for the genera *Bothrops* and *Lachesis muta*, and 1.5 mg/mL for the genus *Crotalus*, is the release of antivenom batches authorized.

However, in recent years, variation has been observed in the quantity of venom produced by the snakes at the CNPB. To analyze this, in a first stage, information published by Zavaleta *et al.*^5^^,^^6^ regarding the population value of snake venom (mg) estimated from the logarithmic regression of venom vs. the logarithm of the number of snakes (PVV) was retrieved. This value was calculated from CNPB production records during the 1970-1986 period, along with the minimum and maximum number of required antivenom serum vials (RAV).

In a second stage, venom production was calculated using CNPB records for the 2013-2023 period, obtained during its routine activities. For this purpose, the weighted mean extraction per venom batch according to species (WMEB) was determined, considering that each batch included a specific number of snakes and an extraction mean. With this information, the range of extraction means (REM) was constructed, corresponding to the minimum and maximum mean of venom extracted in the batches per species.

Based on the REM, the boundary scenarios (lower and upper) of the minimum and maximum estimated quantity of antivenom serum vials (EAV) were constructed, rounded to the next whole value, considering that one vial was required to neutralize 25 mg of venom, as established as the lower limit in the current regulatory framework ^3^. To compare the variation in venom production between that reported by Zavaleta *et al*. ^5^^,^^6^ and the data calculated in the recent period (2013-2023), the absolute and relative difference (%) between PVV and WMEB was obtained (Table 1).

**Table 1:** Mean venom extracted (MVE) per milking and theoretical number of estimated antivenom vials (EAV).

Species	**1970-1986 Records (Taken from Zavaleta *et al*.)** ^(^^5^^,^^6^		2013-2023 Records		
	PVV	RAV	WMEB (REM)	EAV	Diff PVV-WMEB (%)
*Bothrops athrox*	103.8	4 a 11	99.5 (19.7−181.8)	1 a 8	-4.3 (-4.2)
*Bothrops barnetti*	39.1	1 a 3	47.0 (21.4− 87.8)	1 a 4	7.9 (20.1)
*Bothrops pictus*	21.8	1 a 2	28.5 (2.0−46.1)	1 a 2	6.7 (30.6)
*Crotalus durissus*	45.3	2 a 4	68.3 (28.4−147.6)	2 a 6	23 (50.6)
*Lachesis muta*	137.8	5 a 15	211.7 (66.0−368.0)	3 a 15	73.9 (53.6)

PVV: snake venom population value (mg) estimated from the log-log regression of venom vs. the quantity of snakes ^5^^,^^6^, RAV: minimum and maximum number of antivenom serum vials required ^5^^,^^6^, WMEB: weighted average of extraction per venom batch by species, REM: range of the extraction means expressed by the minimum and maximum average of venom extracted in the batches by species, EAV: estimated minimum and maximum number of antivenom serum vials, Diff PVV-WMEB: absolute and relative difference (%) between PVV and WME

As can be seen, the quantity of venom produced by each snake has varied between both periods, a phenomenon already previously described ^7^, which could have important implications for the management of affected patients ^2^. It is proposed that this variation in the quantity of venom produced by snakes in recent years could imply a different number of antivenom vials than those usually required for the management of snakebite poisoning. For example, in the case of *Bothrops atrox*, a 4.2% reduction in venom production was observed; the quantity of vials could theoretically vary from 4 to 11 according to what was reported by Zavaleta *et al.*^5^^,^^6^, whose therapy has been based for some decades now on the use of hyperimmune antivenom serums (antiofídicos), to a range of 1 to 8 based on recent data. Conversely, the species *Lachesis muta* showed a 53.6% increase in the mean venom extracted. Although this did not imply notable variations in the quantity of EAV we estimated compared to what was reported by Zavaleta *et al.*, this could stem from differences in methodology. Nevertheless, all these estimates are purely theoretical and have not been corroborated through clinical studies.

In this context, it is necessary to conduct clinical research that evaluates and validates the quantity of antivenom required for the management of these poisonings ^4^. As it is a public health necessity, it should be considered a priority to be addressed in the short term. Meanwhile, it is important to continue with the production and use of local antivenoms, as there is no substitute for the safety and efficacy offered by an antivenom developed from local herpetofauna, along with medical judgment and patient follow-up to make the respective necessary adjustments.

The experience of some African countries highlights this aspect, as they do not have local antivenom production and must import these products from India and other Asian countries, where they are developed from the venom of different snake species. In many cases, these antivenoms are not effective against poisonings occurring on the African continent, resulting in preventable deaths and disabilities ^8^. In the Latin American context, countries such as Brazil, Colombia, and Costa Rica have modern platforms for antivenom production and are even testing a new generation of antivenoms produced through proteomic techniques, using single-domain antibodies or nanoantibodies. However, these antivenoms are prepared with venoms from snakes present in their respective territories, which are not necessarily the most suitable for treating poisonings that occur in Peru. It is necessary for the country to also advance in the development of these methodologies.

In conclusion, the variation observed in the quantification of venom produced by snakes from the CNPB serpentarium is highlighted, emphasizing the need for clinical studies to update therapeutic recommendations and determine the appropriate doses of antivenom required for the management of snakebite poisonings in our environment.
