# Supplementary material for: Variation in the quantity of venom produced by snakes at the national center for production and strategic public health goods and the need to update antivenom estimations
Source: Rev Peru Med Exp Salud Publica. 2025 Dec 12;42(4):455–7. doi: 10.17843/rpmesp.2025.424.15158 (PMC12879973; doi:10.17843/rpmesp.2025.424.15158)
Supplement: Translation

# Translation: Variación de la cantidad de veneno producido por serpientes en el Centro Nacional de Producción y Bienes Estratégicos de Salud Pública y la necesidad de actualizar las estimaciones de antivenenos

**Authors:** Gualberto Marcas, Gilmer Solis-Sanchez, Flor Fuentes, Leavit López

**Affiliations:** 1 Centro Nacional de Producción y Bienes Estratégicos de Salud Pública, Instituto Nacional de Salud, Lima, Perú.; 2 Centro de Evaluación de Tecnologías en Salud, Instituto Nacional de Salud, Lima, Perú.

Sr. Editor. El envenenamiento por mordedura de serpiente es un problema de salud pública que obedece a diversos factores como la deforestación en la selva, la irrupción de la minería en zonas previamente vírgenes, los cultivos estacionales en ecosistemas tropicales, entre otros. La herpetofauna peruana es particularmente diversa, y la mayoría de envenenamientos por mordedura de serpientes son producidos por *Bothrops atrox* (80%), reportándose con menor frecuencia envenenamiento por mordedura de *Bothrops pictus, Bothrops barnetti, Lachesis muta* y *Crotalus durissus ,* entre otros ^1^.

En el Perú, el manejo de los envenenamientos se ha basado desde hace décadas en antivenenos producidos por el Centro Nacional de Producción y Bienes Estratégicos de Salud Pública (CNPB) del Instituto Nacional de Salud, a partir de suero de equinos inyectados con veneno de serpientes del zoocriadero Oswaldo Meneses. Este centro alberga vipéridos de los tres géneros mencionados, cuyo veneno se extrae mediante un proceso estandarizado ^2^.

Al tratarse de una enfermedad desatendida, son escasos los estudios sobre este tema. Actualmente, la Norma Técnica de Prevención y Tratamiento de Accidentes por Animales Ponzoñosos del año 2005 ^3^ establece las recomendaciones de las dosis de antiveneno que deben utilizarse en el manejo de los pacientes. En ella se indica que el tratamiento específico con suero antiofídico en el Perú se realiza con suero antibotrópico, anticrotálico, y antilachésico con dosis de neutralización de 25 a 40 mg de veneno por frasco ^4^. La potencia del antiveneno se verifica mediante técnicas de neutralización (DE_50_) en ratones y ensayos de dosis letal 50 (DL_50_). Solo después de confirmar que el antiveneno neutraliza 2,5 mg de veneno/ml para los géneros *Bothrops* y *Lachesis muta*, y 1,5 mg/ml para el género *Crotalus*, se autoriza la liberación de los lotes de antivenenos.

Sin embargo, en los últimos años se ha observado variación en la cantidad de veneno producido por las serpientes del CNPB. Para analizar esto, en una primera etapa se recuperó la información publicada por Zavaleta *et al*. ^5^^,^^6^ sobre el valor poblacional de veneno de serpiente (mg) estimado a partir de la regresión logarítmica de veneno *vs.* el logaritmo de cantidad de serpientes (VPV). Este valor se calculó a partir de los registros de producción del CNPB durante el período 1970-1986, junto con la cantidad mínima y máxima de viales de suero antiveneno requeridos (VAR).

En una segunda etapa se calculó la producción de venenos con registros del CNPB correspondiente al período 2013-2023, obtenidos durante sus actividades rutinarias. Para ello, se determinó la media ponderada de extracción por lote de veneno según especie (MPVE), considerando que cada lote incluía un numero determinado de serpientes y una media de extracción. Con esta información se construyó el rango de las medias de extracción (RME), correspondiente a la media mínima y máxima de veneno extraído en los lotes por especie.

A partir del RME, se construyeron los escenarios limítrofes (inferior y superior) de la cantidad mínima y máxima de viales de suero antiveneno estimado (VAE), redondeado al valor entero superior, considerando que se requería un vial para neutralizar 25 mg de veneno, según lo establecido como límite inferior en el marco normativo vigente ^(^^3^. Con el fin de comparar la variación en la producción de veneno entre lo reportado por Zavaleta *et al*. ^5^^,^^6^ los datos calculados en el periodo reciente (2013-2023), se obtuvo la diferencia absoluta y relativa (%) entre VPV y la MPVE (tabla 1).

**Tabla 1:** Media de veneno extraído (MVE) por ordeño y numero teórico de viales antiveneno estimado (VAE).

Especie	Registros de 1970-1986 (Tomado de Zavaleta *et al.*) ^5^^,^^6^		Registros de 2013-2023		
	VPV	VAR	MPVE (RME)	VAE	Dif VPV-MPVE (%)
*Bothrops athrox*	103,8	4 a 11	99,5 (19,7−181,8)	1 a 8	-4,3 (-4,2)
*Bothrops barnetti*	39,1	1 a 3	47,0 (21,4− 87,8)	1 a 4	7,9 (20,1)
*Bothrops pictus*	21,8	1 a 2	28,5 (2,0−46,1)	1 a 2	6,7 (30,6)
*Crotalus durissus*	45,3	2 a 4	68,3 (28,4−147,6)	2 a 6	23 (50,6)
*Lachesis muta*	137,8	5 a 15	211,7 (66,0−368,0)	3 a 15	73,9 (53,6)

VPV: valor poblacional de veneno de serpiente (mg) estimado a partir de la regresión logarítmica de veneno vs. el logaritmo de cantidad de serpientes ^5^^,^^6^, VAR: cantidad mínima y máxima de viales de suero antiveneno requeridos ^5^^,^^6^, MPVE: media ponderada de extracción por lote de veneno según especie, RME: rango de las medias de extracción expresada por la media mínima y máxima de veneno extraído en los lotes por especie, VAE: cantidad mínima y máxima de viales de suero antiveneno estimado, Dif PVR-MPVE: diferencia absoluta y relativa (%) entre VPV y MPVE.

Como se puede apreciar, la cantidad de veneno producida por cada serpiente ha variado entre ambos periodos, un fenómeno ya descrito previamente ^7^, lo cual podría tener importantes implicancias para el manejo de los pacientes afectados ^2^. Se plantea que esta variación de la cantidad de veneno producido por las serpientes en los últimos años podría implicar una cantidad de viales de antivenenos distinta a la habitualmente requerida para el manejo del envenenamiento por mordedura de serpiente. Por ejemplo, en el caso de *Bothrops athrox*, se observó una reducción de 4,2% en la producción de veneno, la cantidad de viales teóricamente podría variar de 4 a 11 según lo reportado por Zavaleta *et al*. ^5^^,^^6^^)^ y su terapéutica se basa desde algunas décadas atrás, en el empleo de los sueros hiperinmunes antiveneno (antiofídicos, a un rango de 1 a 8 con base en los datos recientes. Por el contrario, la especie *Lachesis muta* presentó un incremento de 53,6% en la media de veneno extraído. Aunque esto no implicó variaciones notables en la cantidad de VAE que estimamos respecto a lo reportado por Zavaleta *et al.*, lo cual podría derivar de las diferencias en la metodología. No obstante, todas estas estimaciones son puramente teóricas y no han sido corroboradas mediante estudios clínicos.

En ese contexto, resulta necesario realizar investigación clínica que evalúe y valide la cantidad de antiveneno requerida para el manejo de estos envenenamientos ^4^. Siendo una necesidad de salud pública, debería considerarse una prioridad a ser atendida en el corto plazo. Mientras tanto, es importante continuar con la producción y uso de antivenenos locales, ya que no existe sustituto para la seguridad y eficacia que ofrece un antiveneno elaborado a partir de la herpetofauna local, junto con el criterio médico y el seguimiento del paciente para realizar los ajustes respectivos necesarios.

La experiencia de algunos países africanos resalta este aspecto, ya que, al no contar con producción local de antivenenos, deben importar estos productos desde la India y otros países asiáticos, donde se elaboran a partir del veneno de especies distintas de serpientes. En muchos casos, estos antivenenos no resultan efectivos frente a los envenenamientos ocurridos en el continente africano, lo que genera muertes y discapacidades evitables ^8^. En el contexto latinoamericano, países como Brasil, Colombia y Costa Rica disponen de plataformas modernas para la producción de antivenenos, e incluso están probando una nueva generación de antivenenos producidos mediante técnicas proteómicas, utilizando anticuerpos de dominio único o nanoanticuerpos. Sin embargo, dichos antivenenos se elaboran con venenos de serpientes presentes en sus respectivos territorios, los cuales no necesariamente son los más adecuados para tratar los envenenamientos que ocurren en el Perú. Es necesario que el país también avance en el desarrollo de estas metodologías.

En conclusión, se destaca la variación observada en la cuantificación del veneno producido por serpientes del serpentario del CNPB, lo cual enfatiza la necesidad de realizar estudios clínicos que permitan actualizar las recomendaciones terapeuticas y determinar las dosis adecuadas de antiveneno que se requieren para el manejo de los envenenamientos por mordedura de serpiente en nuestro medio.
